# Systemic propranolol and topical moist wound dressings for ulcerated infantile hemangioma of the scrotum: a case report

**DOI:** 10.3389/fped.2026.1752616

**Published:** 2026-05-04

**Authors:** Jiejun Xia, Kunshan Chen, Zhenyin Liu, Xiaoyun Tan, Shifeng Xie, Haibo Li

**Affiliations:** Department of Interventional Radiology and Vascular Anomalies, Guangzhou Women and Children’s Medical Center, Guangzhou Medical University, Guangzhou, China

**Keywords:** alginate dressing, case report, moist wound healing, propranolol, scrotal hemangioma, ulcerated infantile hemangioma

## Abstract

**Background:**

Ulceration is a common complication of infantile hemangioma, and its management poses unique difficulties when the lesion is located in high-risk areas like the scrotum. This case report details the successful management of this condition using topical moist wound dressings combined with propranolol.

**Case presentation:**

A 5-month-old male presented with a rapidly progressing, ulcerated scrotal infantile hemangioma characterized by purulent exudate. Systemic treatment involved oral propranolol solution (maintenance dose of 2 mg/kg/d). Local wound care was managed using a tailored moist wound healing strategy: alginate dressing combined with recombinant human epidermal growth factor gel, secured with a transparent film dressing. The patient was treated successfully; localized infection was controlled within 3 days. The wound achieved complete epithelialization after a total of 12 days of consistent combined treatment, accompanied by simultaneous regression of the hemangioma volume. Three-month Follow-up confirmed favorable cosmetic results with no significant hypertrophic scarring.

**Conclusion:**

This case underscores that a synergistic regimen of systemic propranolol and a customized moist wound healing strategy can significantly accelerate healing timelines and improve the quality of tissue repair in ulcerated infantile hemangiomas.

## Introduction

Infantile Hemangioma (IH) is the most common benign tumor of infancy, exhibiting a predilection for females and preterm infants. While they most frequently occur in the head and neck, lesions in the anogenital region are considered high-risk due to complications such as ulceration, bleeding, and secondary infection. Standard treatments include systemic *β*-blockers, topical agents, and laser therapy (1). Although the majority of IH spontaneously involute, approximately 15% of cases develop ulceration, known as ulcerated infantile hemangioma (UIH) (2). UIHs may present a clinical challenge. This report details the successful management of a scrotal UIH using combined propranolol and a customized MWH dressing regimen, providing a reference for achieving rapid healing in complex anatomical sites.

## Case presentation

A 5-month-old male infant was admitted to our department for the evaluation and management of a complicated UIH located on the scrotum. The patient had an unremarkable perinatal history and had been generally healthy prior to this complaint. The scrotal mass was first noted shortly after birth and initially exhibited a typical pattern of uncomplicated rapid growth. However, the clinical course deteriorated one month prior to admission when the lesion developed surface ulceration accompanied by intermittent bleeding. Although the patient had received topical treatments at a local clinic, the condition showed no signs of improvement and progressed to secondary infection.

**Figure 1:**
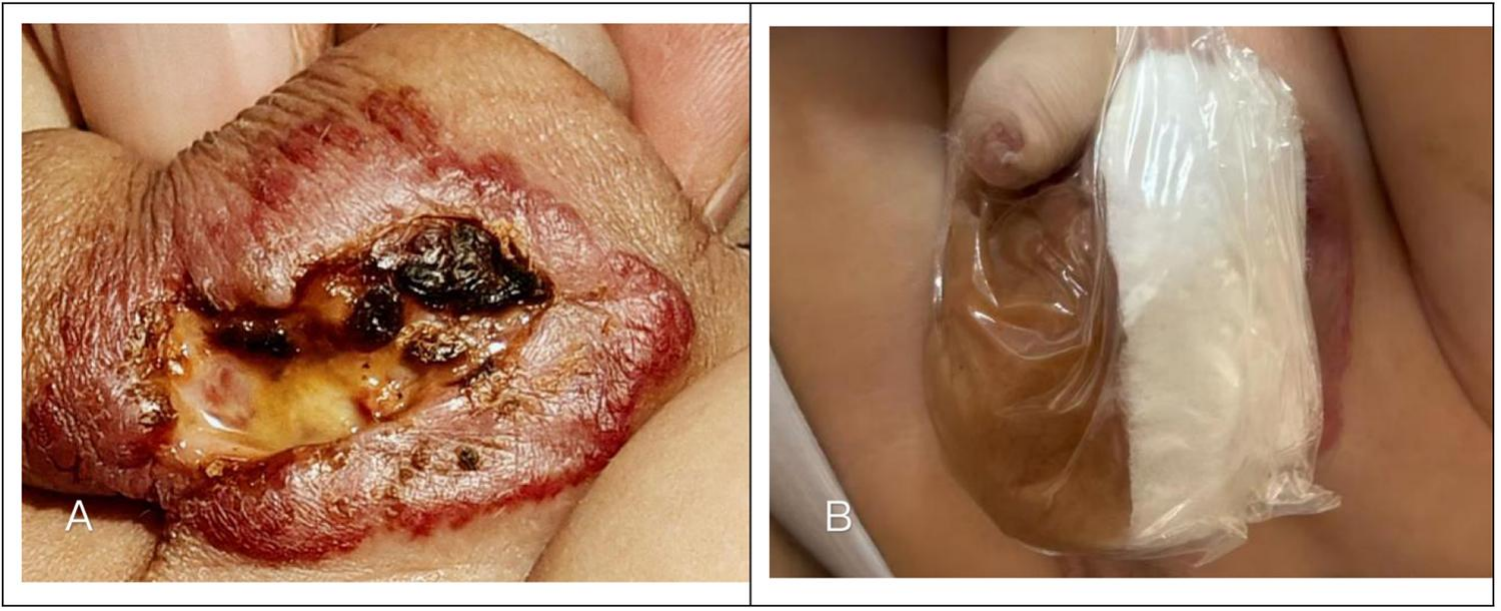
Clinical presentation and initial treatment of the scrotal ulcerated infantile hemangioma. **(A)** The large red mass on the scrotum upon the patient's admission, showing a visible ulcerated area with purulent discharge. **(B)** Application of topical Moist Wound Healing (MWH) dressings.

Upon admission, the systemic physical assessment revealed a well-nourished infant with stable vital signs; he was afebrile and displayed normal feeding and sleeping patterns. In contrast, the local examination of the genitalia revealed a substantial erythematous soft-tissue mass encompassing the scrotum. A central ulcerative defect measuring approximately 2 × 3 cm was evident on the hemangioma's surface. The ulcer crater extended 0.3 cm into the tissue and in contrast to clean wounds, the ulcer base was obscured by purulent exudate and necrotic debris (Figure 1A). No other congenital anomalies were detected during the survey. Baseline laboratory investigations, specifically an electrocardiogram (ECG), were performed to assess suitability for beta-blocker therapy. The ECG revealed a normal sinus rhythm with no conduction abnormalities, confirming that the patient was suitable for beta-blocker therapy.

Following the exclusion of contraindications and a thorough discussion with the parents regarding the risks, benefits, and potential outcomes of various therapeutic options, a shared decision was made to proceed with a multimodal strategy. Systemic pharmacotherapy commenced with oral propranolol solution at a starting dosage of 1 mg/kg/day. The initial administration was performed in a day-care ward setting, where the patient was monitored with continuous electrocardiography for 2 h. After ensuring stable vital signs and the absence of adverse cardiovascular events, the patient was discharged on the same day. The dosage was gradually titrated to a maintenance dosage of 2 mg/kg/day for outpatient care. Concurrently, an aggressive local moist wound healing (MWH) protocol was established to address the infection and exudate. This regimen involved once-daily debridement with iodophor, followed by the application of recombinant human epidermal growth factor (rhEGF) gel to stimulate tissue regeneration. To manage the heavy exudate, an alginate dressing was applied and meticulously secured with a 3M transparent film to create a waterproof barrier against urine contamination (Figure 1B).

Given the necessity for consistent ongoing care, the parents received comprehensive training on this specific aseptic dressing technique. Upon mastering the procedure, they assumed responsibility for the daily wound care at home. Furthermore, to ensure close surveillance of the healing trajectory without requiring daily hospital visits, the parents were instructed to capture high-resolution digital photographs of the wound during each dressing change and transmit them directly to the attending physician for real-time evaluation.

**Figure 2:**
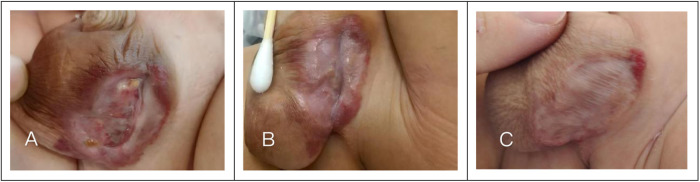
Clinical progression and healing of the scrotal ulcerated infantile hemangioma during combined therapy. **(A)** Wound condition after 3 days of treatment: Epithelialization is visible at the wound edges, with vigorous granulation tissue growth in the center. **(B)** Wound condition after 12 days of treatment: The ulcerated area has achieved complete epithelialization. **(C)** Wound healing progression and final outcome after 3 months.

The clinical response to this managed care was rapid: the purulent discharge ceased, revealing healthy granulation tissue within 3 days (Figure 2A). Continuous combined therapy led to marked tumor regression and complete epithelialization of the ulcer within 12 days (Figure 2B). The patient was subsequently discharged on a 3-month propranolol maintenance plan, with follow-up assessments confirming a satisfactory cosmetic result devoid of hypertrophic scarring (Figure 2C).

The clinical course and key interventions are summarized in Table 1.

**Table 1:** Chronological timeline of clinical events, interventions, and outcomes for the patient with scrotal ulcerated infantile hemangioma.

Time	Events and Interventions
Birth	Uncomplicated erythematous mass noted on the scrotum.
4 Months	Developed surface ulceration with intermittent bleeding.
5 Months (Day 0)	Admission: Scrotal UIH (2 × 3 cm) with infection Treatment: Oral propranolol (2 mg/kg/d) + daily MWH dressing.
Day 3	Infection controlled; healthy granulation tissue appeared.
Day 12	Complete epithelialization achieved; marked tumor regression.
3 Months	Satisfactory cosmetic result; no hypertrophic scarring.

### Patient perspective

The parents were initially distressed by the bleeding and secondary infection of the scrotal lesion. After receiving training on aseptic dressing techniques, they felt confident in managing the daily wound care at home. They expressed high satisfaction with the 12-day complete healing and the final cosmetic outcome, which exceeded their expectations.

## Discussion

The management of ulcerated infantile hemangioma (UIH) presents a major clinical challenge due to associated morbidities, including pain, bleeding, secondary infection, and the risk of permanent scarring or disfigurement (3, 4). The lesion in this case was located on the scrotum, a part of the diaper anogenital area, which is classified as a high-risk location. Ulcerations in this region are particularly difficult to manage as they are continuously exposed to friction, urine, and stool, making them prone to infection and recalcitrance to healing (5). The patient presented with active ulceration, intermittent bleeding, and secondary infection with purulent exudate, necessitating prompt and aggressive multi-modal intervention. While surgical excision is an option discussed in the literature for scrotal hemangiomas, primarily due to concerns regarding potential testicular damage and fertility, the majority of UIH management relies on medical therapy combined with stringent wound care (6).

Systemic propranolol therapy has become the first-line treatment for complicated and ulcerated infantile hemangiomas. Its efficacy in promoting rapid ulcer healing is well-established; a systematic review reported that 191 out of 197 patients (97.0%) treated with oral propranolol achieved complete ulcer healing (5). However, despite the widespread use of *β*-blockers, a subset of patients with UIH still experiences prolonged healing times. In a large retrospective multicenter cohort study, the median time to heal for patients receiving systemic *β*-blockers was 6.36 weeks, and for those receiving multimodal therapy, it was 7.71 weeks (5). The exceptional healing time observed in our patient—12 days (approximately 1.7 weeks)—is significantly faster than these median times. This rapid result aligns with some of the quickest outcomes reported in the literature, such as an average healing time of 17.9 days observed in one prospective study of propranolol treatment (7).

In addition to systemic treatments, topical solutions such as timolol have been increasingly recognized for their efficacy in aiding UIH closure with a favorable safety profile. Recent literature, including a comparable case of anogenital UIH successfully managed with topical timolol (8), highlights its potential to mitigate the extra risks associated with systemic oral *β*-blockers. However, in our specific case, the scrotal lesion presented with a relatively deep ulcer crater extending 0.3 cm into the tissue. Given the concern that topical agents might not achieve adequate tissue penetration to the base of such a deep lesion, we opted for systemic propranolol combined with a rigorous local dressing regimen to ensure robust and comprehensive therapeutic coverage.

Beyond systemic control, wound care is the cornerstone of UIH management. Almost invariably, all patients with IH ulceration receive some form of wound care (9). Current literature reports various wound care strategies for UIH, including the use of barrier creams, non-adherent silicone dressings, antimicrobial dressings, and pulsed dye laser (PDL) therapy, which aim to reduce pain and promote re-epithelialization (10). The maintenance of a moist wound environment is paramount, as it facilitates autolytic debridement (essential for clearing necrotic debris), accelerates keratinocyte migration, and reduces pain by preventing the desiccation of nerve endings and avoiding adherence to the wound bed (11).

The rapid re-epithelialization in this challenging anogenital location underscores the crucial role of meticulous home care. While systemic therapy provides structural tumor control, achieving a swift 12-day outcome in such a high-contamination area reflects the parents’ strict adherence to the daily wound care regimen. Ultimately, parental education and compliance are critical for optimizing local wound conditions and maximizing the benefits of systemic therapy in UIH.

## Strengths and limitations

A major strength of this report is the rapid 12-day healing of a high-risk scrotal UIH using a non-invasive, parent-led multimodal strategy. This provides a safe alternative to surgery. However, the study is limited by being a single case report, and the success highly depends on strict parental compliance and proficiency in wound care. Further studies with larger cohorts and longer follow-up are needed to validate these findings.

## Conclusion

This case of complex scrotal UIH demonstrates that the synergistic use of systemic propranolol therapy combined with rigorous, parent-executed wound management can lead to markedly accelerated healing compared to average reported timelines. Furthermore, it highlights that thorough instruction and education of parents regarding wound care principles are indispensable for achieving rapid and successful resolution of ulcerated infantile hemangiomas, particularly those located in high-risk areas.

## Data Availability

The original contributions presented in the study are included in the article/Supplementary Material, further inquiries can be directed to the corresponding author.
